# The abiding, hidden, and pervasive centrality of the health research workforce

**DOI:** 10.1186/s12960-023-00821-9

**Published:** 2023-05-11

**Authors:** Paulo Ferrinho, Michael Makanga, Shabnum Sarfraz, Mario Dal Poz

**Affiliations:** 1 Global Health and Tropical Medicine (GHTM), Institute of Hygiene and Tropical Medicine, NOVA University of Lisbon, 1349-008 Lisbon, Portugal; 2 European & Developing Countries Clinical Trials Partnership (EDCTP), The Hague, The Netherlands; 3 P2Impact Associates Pakistan, Women in Global Health, Harvard Global Health Institute, Cambridge, United States of America; 4 Institute of Social Medicine (IMS), University of the State of Rio de Janeiro (UERJ), Rio de Janeiro, Brazil

**Keywords:** Research for health, Health research workforce, Health researchers’ labour market

## Abstract

Research for health and development (R4HD) acknowledges that many of the determinants of health lie outside the boundaries of the health system. The size and quality of the health and care workforce (HCWF) are key drivers towards the future trajectory of many of these factors. We consider researchers for health and development an abiding, pervasive but neglected constituent part of this HCWF. This workforce straddles many professional groups and sectors. The diversity of occupations, lack of standardization in occupational cadres, the complexity and gendered aspects of the labour market, and the variable demographic, epidemiological, socio-economic and health systems’ contexts in the global south and the global north, led to a kaleidoscopic perception of the health research workforce that have kept it hidden from public opinion. This led to neglect by science as well as health policymakers and created an orphan sub-set of the HCWF. Understanding the health researchers’ labour market will help to identify means to develop, retain and utilize the health research workforce, addressing size, composition, role, skills transferability, careers and social impact through building, enabling or sustaining its research functions, capacity, employment opportunities and career tracks, among other issues. This thematic series of the Human Resources for Health Journal, calls for papers that go beyond narrow conceptual approaches and professional understandings of health care workers and the health research workforce, and requests that contributors examine important workforce issues through the broad lens of R4HD within a sustainable development goals framework.

## Editorial

Research for health and development (R4HD) acknowledges that many of the determinants of health, such as the social and economic environment, the physical ecosystem, and individual characteristics and behaviours, lie outside the boundaries of the health system [1]. However, the size and quality of the health and care workforce (HCWF) as champions for health, wellbeing, development and research, are key drivers towards the future trajectory of many of these factors. We consider researchers for health and development an abiding, pervasive but neglected constituent part of this HCWF.

R4HD is a critical component of improving health and equity and of achieving Universal Health Coverage (UHC) [1–4]. Good R4HD requires robust and sustainable national research systems. National health research systems (NHRS) serve as key components of a society’s ability to respond to both acute and long-term health needs [1]. As such, NHRS integrate both national research systems and national health systems and can be defined “as the people, institutions and activities whose primary purpose is to generate and promote the utilisation of high quality scientific knowledge” to encourage “its utilisation in strengthening national health systems to be responsive, to provide social and financial risk protection, to improve efficiency of services, and, ultimately, to improve the health of the population” and the planet [5].

Strengthening NHRS has received a lot of attention over the past two decades [6–10], with a focus on three essential pillars: governance/stewardship, developing and sustaining assets and producing and using R4HD [2]. Although organizations with technical mandates in or related to R4H report being engaged in all pillars, the most intense activity has been in relation to governance and using health research [11].

The assets pillar includes a competent, stable, well-resourced, motivated and multifaceted health research workforce for biomedical, bioscience, epidemiology, health, public health, global health, human rights, social sciences and health systems research. This workforce straddles many professional groups and sectors—the International Standard Classification of Occupations’ (ISCO 08) Index of Occupational Titles, adopted by the International Labour Organization in 2007, lists numerous potentially research-related occupations under “academics”, “biostatisticians”, “epidemiologists”, “researchers”, “scientists” and several biological, biomedical and health-related professions and occupations (https://www.ilo.org/public/english/bureau/stat/isco/isco08/index.htm).

As such, the labour market for the health research workforce includes universities, research institutes, state departments and ministries, hospitals and other health facilities, public health institutes, pharmaceutical industry, consultancy firms, multilateral development agencies and civil society organizations [2, 11]. The diversity of occupations, lack of standardization in occupational cadres, the complexity and gendered aspects of the labour market, and the variable demographic, epidemiological, socio-economic and health systems’ contexts in the global south and the global north, led to a kaleidoscopic perception of the health research workforce that have kept it hidden from public opinion.

This in turn has led to neglect by science as well as health policymakers and created an orphan sub-set of the HCWF, ignored in human resources for health-related global health initiatives, overlooked by health and policies, absent from national health strategies, unacknowledged in national health workforce plans and unrecognized /discouraged in career paths of health care providers. The silo mentality of the different governance sectors contributes to the lack of dialogue that prevents multisectoral policies and plans to address the issue.

R4HD demands multisectoral attention and efforts. Many of the critical issues in this area are cross-disciplinary in nature, have been discussed conceptually but remain short of a unified conceptual viewpoint and neglect practical implementation bottlenecks, such as the workforce issues across many social sub-systems (e.g. science and technology sub-system, health and care sub-system, industrial sub-system, education sub-systems) in a complex, ill-defined labour market, described by some as an “ecosystem in structural disequilibrium”[12].

The causes of this disequilibrium vary among high-income (HIC), middle-income (MIC) and low-income countries (LIC) [13, 14]. HIC have gone through “boom and bust cycles during the millennium transition”, associated with expansion of funding opportunities that flattened with emerging budget constraints towards the end of the first decade of the new millennium, creating an unsustainable increase of the health research workforce [12]. Opportunities in LIC are growing fast, but not as fast as the health research workforce, creating a paradoxical situation of scarcity of resources associated with abandonment of the research career, emigration or unemployment. In both contexts, for different reasons, supply consistently outstrips demand.

Understanding the health researchers’ labour market will help to identify means to develop, retain and utilize the health research workforce, addressing size, composition, role, skills transferability, careers and social impact through building, enabling or sustaining its research functions, capacity, employment opportunities and career tracks, among other issues [15].

There remains a large gap in our understanding of these "hidden" health workers. Their abiding centrality became apparent during the SARS-CoV2 pandemic. This thematic series of the Human Resources for Health Journal, calls for papers that go beyond narrow conceptual approaches and professional understandings of health care workers and the health research workforce, and requests that contributors examine important workforce issues through the broad lens of R4HD within a sustainable development goals framework.

The articles for this thematic series should link to equity issues and bring in an international cooperation angle including south–south, north–south, north–north and triangular collaborations and/or other mechanisms to optimize development, retention and sustainability of the health research workforce.

Full details of the Call are at: The deadline for submissions is 31st December 2023. Manuscripts must follow the Journal Guidelines for contributors available at https://human-resources-health.biomedcentral.com/submission-guidelines and mention this call for papers in the cover letter. All submissions will be reviewed by peers.

## Data Availability

Not applicable.
